# Diffusion Tensor Imaging Along the Perivascular Space (DTI-ALPS) as a Neuroimaging Biomarker of Glymphatic Function in Neurodegenerative Diseases: A Systematic Review

**DOI:** 10.3390/ijms27135758

**Published:** 2026-06-26

**Authors:** Raphael Lopes Olegário, Otávio Toledo Nóbrega, Naiara Ribeiro Almeida, Dany Alexis Sobarzo Soto, Ciro José Brito, Diógenes Diego de Carvalho Bispo, Felipe von Glehn, Arsenio Páez, Thien Thanh Dang-Vu, Einstein Francisco Camargos

**Affiliations:** 1 Faculty of Medicine, University of Brasília (UnB), Brasília 70910-900, Brazil; contact@rlolegario.com (R.L.O.); otavionobrega@unb.br (O.T.N.); dbispo.neurorradio@gmail.com (D.D.d.C.B.); felipe.vonglehn@unb.br (F.v.G.); 2 Department of Clinical Medicine, Geriatric Medicine Centre, University Hospital of Brasília, University of Brasília (UnB), Brasília 70910-900, Brazil; 3 Department of Physical Education, Federal University of Juiz de Fora, Juiz de Fora 36036-900, Brazil; naiara.ribeiro@ufjf.br (N.R.A.); danysobarzo@santotomas.cl (D.A.S.S.); ciro.brito@ufjf.br (C.J.B.); 4 Escuela de Kinesiologia, Universidad Santo Tomás, Puerto Montt 5480000, Chile; 5 Diagnostic Imaging Unit, University Hospital of Brasília, Brasília 70840-901, Brazil; 6 Department of Health, Kinesiology and Applied Physiology, Concordia University, Montreal, QC H4B 1R6, Canada; arsenio.paez@concordia.ca (A.P.); tt.dangvu@concordia.ca (T.T.D.-V.); 7 Centre de Recherche de l’Institut Universitaire de Gériatrie de Montréal (CRIUGM), Montreal, QC H3W 1W5, Canada

**Keywords:** glymphatic system, DTI-ALPS, Alzheimer’s disease, neuroimaging, biomarkers

## Abstract

The glymphatic system has been proposed as a brain-wide pathway that promotes the exchange between cerebrospinal and interstitial fluids and facilitates the clearance of metabolic waste products, including amyloid-β and tau proteins. Diffusion tensor imaging analysis along the perivascular space (DTI-ALPS) has emerged as a non-invasive magnetic resonance imaging technique proposed to indirectly assess glymphatic-related fluid dynamics. This systematic review evaluated the methodological consistency and clinical applicability of the ALPS index in neurodegenerative diseases. A structured search of PubMed (MEDLINE) and Web of Science identified human studies published up to January 2026 investigating DTI-ALPS in neurodegenerative conditions. Data regarding study populations, MRI acquisition parameters, image-processing methods, statistical approaches, and clinical associations were extracted and synthesized. Ten studies met the inclusion criteria. Across studies, lower ALPS index values were generally associated with cognitive impairment, amyloid burden, and disease severity, particularly in Alzheimer’s disease. Several studies incorporated multimodal biomarkers, including amyloid positron emission tomography and cerebrospinal fluid markers, thereby improving the biological interpretation of DTI-ALPS findings. However, substantial methodological heterogeneity was identified across studies, including variability in region-of-interest placement, diffusion acquisition protocols, and image-processing pipelines. Furthermore, the interpretation of diffusivity metrics as direct measures of glymphatic flow remains controversial. Current evidence suggests that DTI-ALPS may represent a promising non-invasive imaging marker of glymphatic-related alterations; however, its biological specificity and clinical applicability remain insufficiently established. Standardized acquisition protocols, harmonized analytical pipelines, and longitudinal multicenter studies are required to clarify its role in neurodegenerative disease research.

## 1. Introduction

The absence of a conventional lymphatic system in the brain has led to the proposal of the glymphatic system (GS), a brain-wide pathway that facilitates the exchange between cerebrospinal fluid (CSF) and interstitial fluid (ISF), thereby supporting the clearance of extracellular solutes, including neurotoxic proteins such as amyloid-β (Aβ) and tau [1]. The GS is thought to play a crucial role in clearing waste from the central nervous system (CNS), and its activity is modulated by various physiological factors, particularly sleep [2].

Most evidence supporting glymphatic function derives from animal models, and no gold-standard method currently exists for directly measuring GS activity in humans. To address this limitation, researchers have adopted existing neuroimaging techniques, most notably magnetic resonance imaging [3], to evaluate features such as the perivascular space. Advancements in neuroimaging have enabled non-invasive evaluation of GS processes, particularly through diffusion tensor image analysis along the perivascular space (DTI-ALPS), which quantifies water diffusion along perivascular pathways as an indirect marker of glymphatic function without requiring contrast agents [4]. Although the glymphatic model has gained substantial attention in recent years, its applicability to the human brain remains debated. Some investigators argue that diffusion-driven transport mechanisms may predominate over large-scale convective bulk flow, whereas others support the existence of organized perivascular clearance pathways. Accordingly, biomarkers proposed to reflect glymphatic activity should be interpreted cautiously until further physiological validation becomes available.

**Figure 1:**
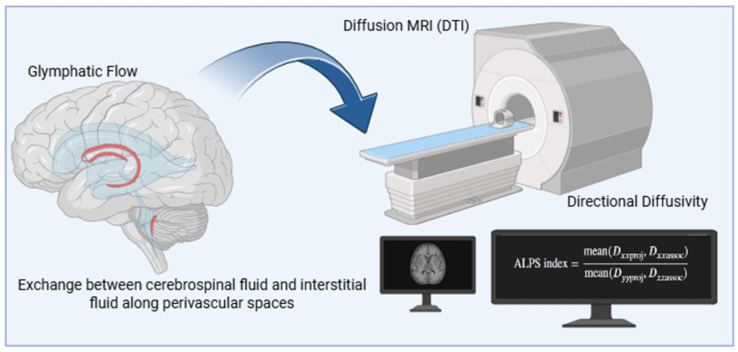
Conceptual relationship between glymphatic system function and the DTI-ALPS approach. Created in Biorender. Olegário et al. (2026). Note: The ALPS index reflects directional diffusivity patterns associated with perivascular spaces but does not directly measure glymphatic flow; its interpretation should therefore be considered as an indirect approximation of underlying fluid transport processes.

The relationship between glymphatic fluid dynamics and the DTI-ALPS approach can be conceptually understood by considering the interaction between cerebrospinal fluid–interstitial fluid exchange and directional water diffusivity measured by diffusion tensor imaging (Figure 1).

The ALPS index is derived from directional diffusivity measurements obtained in white matter regions where projection fibers and association fibers run perpendicular to medullary veins and presumed perivascular spaces [5]. Under this framework, increased diffusivity parallel to the perivascular orientation is hypothesized to reflect greater fluid mobility along these spaces. However, DTI metrics do not directly quantify CSF or convective transport. Instead, they reflect the movement of water molecules within tissue microenvironments and are influenced by multiple structural and physiological factors, including fiber orientation, extracellular space composition, vascular pulsatility, inflammation, edema, and tissue degeneration [6,7]. Consequently, the interpretation of ALPS measurements as direct indicators of glymphatic flow remains controversial. Based on these directional diffusivity measurements, the ALPS index is computed as follows:

$$\frac{\left(D_{\left(xx\right)}^{\left(proj\right)}+D_{\left(xx\right)}^{\left(assoc\right)}\right)}{\left(D_{\left(yy\right)}^{\left(proj\right)}+D_{\left(zz\right)}^{\left(assoc\right)}\right)}$$

$D_{\left(xx\right)}^{\left(proj\right)}$ and $D_{\left(xx\right)}^{\left(assoc\right)}$ correspond to diffusivities measured along the *x*-axis in projection and association fiber regions, respectively, whereas $D_{\left(yy\right)}^{\left(proj\right)}$ and $D_{\left(zz\right)}^{\left(assoc\right)}$ correspond to diffusivities measured perpendicular to the presumed perivascular space direction in projection and association fiber regions. The ratio provides an indirect measure of diffusivity along the perivascular space. In this framework, diffusivity components aligned with the presumed orientation of perivascular spaces are compared with diffusivity components perpendicular to major white matter tracts, generating an indirect index of perivascular diffusivity patterns.

In animal studies, the GS is primarily studied in vivo using advanced imaging techniques such as two-photon microscopy and tracer-based methods, in which fluorescent or radiolabeled molecules are introduced into the CSF to visualize its movement through the brain [8]. These approaches provide high spatial and temporal resolution but are invasive and not feasible in humans due to ethical and technical constraints, including the requirement for surgical access and risks associated with intrathecal or intracerebral tracer administration [8].

These approaches permit direct visualization of tracer movement with high spatial and temporal resolution but are not routinely applicable in humans because of ethical and technical limitations. Consequently, non-invasive neuroimaging methods have emerged as indirect alternatives for investigating putative glymphatic-related processes in vivo.

Among emerging non-invasive approaches to assess glymphatic function, DTI-ALPS has gained increasing attention. The ALPS (Analysis along the Perivascular Space) index, derived from DTI, quantifies water diffusion along perivascular spaces and has been used as a proxy for glymphatic activity. It provides an indirect measure of fluid transport without the need for contrast agents. In Alzheimer’s disease (AD), lower ALPS indices have been associated with reduced perivascular diffusivity and poorer cognitive performance, supporting its potential utility as a biomarker of glymphatic dysfunction [5].

Unlike conventional diffusion tensor imaging metrics such as fractional anisotropy (FA) and mean diffusivity (MD), which primarily reflect tissue microstructural organization and overall diffusivity, the ALPS index was specifically developed to evaluate directional diffusivity patterns adjacent to presumed perivascular spaces. Free-water imaging, in contrast, estimates extracellular water content and tissue compartmentalization. Therefore, although these diffusion-derived metrics may share biological association, they represent distinct physiological and analytical constructs and should not be interpreted interchangeably.

This review aims to evaluate the consistency, methodological variability, and clinical applicability of the ALPS index as a non-invasive neuroimaging biomarker of GS function. We synthesize evidence from clinical studies to characterize its strengths, limitations, and reproducibility in detecting glymphatic dysfunction, particularly in neurodegenerative diseases. We discuss methodological considerations, potential confounding factors, and the extent to which the ALPS index reflects underlying glymphatic activity.

## 2. Methods

A systematic literature search was conducted in two electronic databases, PubMed (via MEDLINE) and Web of Science Core Collection (WoSCC), to identify studies published up to January 2026. The search strategy incorporated relevant Medical Subject Headings (MeSH) terms and keywords related to DTI-ALPS and glymphatic system (GS) assessment in humans. The full search strategy is provided in Appendix A.

Eligibility criteria were defined a priori. In cases where multiple studies met the eligibility criteria, priority was given to those providing complete methodological descriptions, clearly defined regions of interest, and statistical analyses. This approach was adopted to enhance comparability across studies and ensure the inclusion of methodologically consistent datasets. Studies were included if they were peer-reviewed original research in human populations and used DTI-ALPS to assess GS-related processes. Eligible studies focused on mild cognitive impairment (MCI), AD, or other neurodegenerative diseases and were required to report diffusion tensor metrics relevant to the GS (e.g., FA, MD, and/or the ALPS index). Studies combining DTI-ALPS with other imaging modalities (e.g., contrast-enhanced magnetic resonance imaging (MRI), functional MRI, or CSF flow imaging) were included when relevant to the review objectives.

After the database search, records were exported to Microsoft Excel and processed in RStudio (version 2024.12.0, Build 467). Duplicates were removed, and titles/abstracts were screened using manual review complemented by scripted filtering in R. Data handling and text processing used dplyr (wrangling/filtering), stringr (text cleaning and pattern matching), tidyr (management of structured text fields), and readr (file import/export).

Data extraction was performed using a structured table capturing methodological and analytical characteristics, including sample size and clinical groupings, MRI field strength (1.5T or 3T), DTI acquisition parameters (e.g., b-values and number of diffusion directions), and diffusion metrics (e.g., ALPS index, FA, and MD).

The methodological quality and risk of bias of the included studies were assessed using the Joanna Briggs Institute (JBI) Critical Appraisal Tool for analytical cross-sectional studies (OSF repository). Two reviewers independently evaluated each study, and discrepancies were resolved through consensus or consultation with a third senior reviewer.

Data were synthesized using qualitative and quantitative approaches. Descriptive statistics summarized study characteristics, imaging protocols, and key findings. Data processing and visualization were conducted in R (v3.6.2), using ggplot2 (v3.2.1) for publication-quality bar charts and patchwork/gridExtra for figure assembly. The study-selection flow diagram was created using ggplot2 and grid to approximate a PRISMA-style layout, and an additional version was generated with DiagrammeR (Graphviz) for interactive visualization.

## 3. Results and Discussion

The literature search identified studies investigating the glymphatic system using DTI-ALPS in human populations, focusing on neurodegenerative conditions such as AD and mild cognitive impairment, and published between 2010 and January 2026. Only original peer-reviewed studies reporting ALPS-related diffusion metrics were considered.

**Figure 2:**
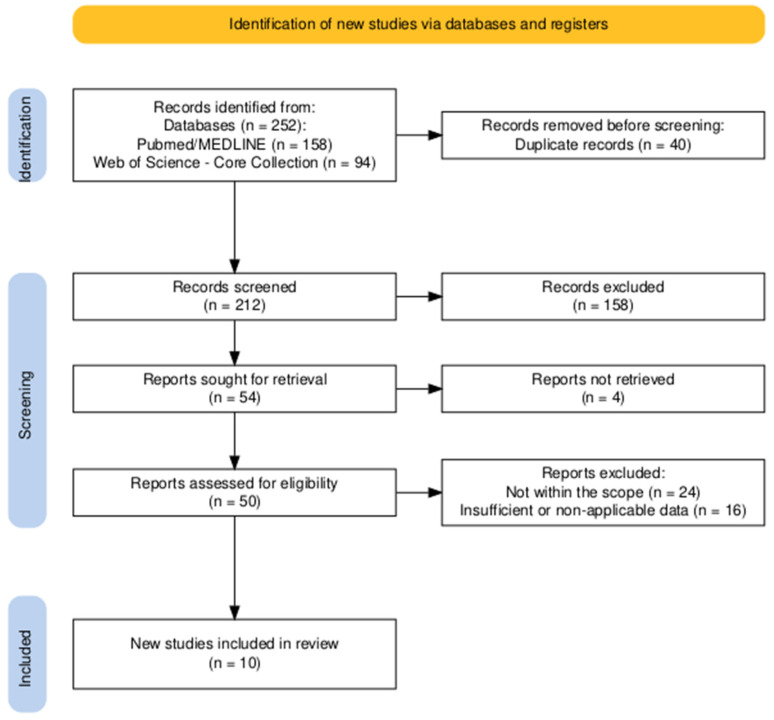
PRISMA flow diagram of study selection and inclusion process.

A total of 252 records were identified, including 158 from PubMed (via MEDLINE) and 94 from the Web of Science Core Collection (WoSCC). After removing 40 duplicates using a combination of Excel and RStudio, 212 unique articles remained for initial screening. Titles and abstracts were assessed using a hybrid approach that combined manual review with automated keyword filtering. Of the 50 potentially eligible studies, 10 met all predefined inclusion criteria and were retained for full-text review and data extraction. Studies were excluded if they (i) did not report the ALPS index or relevant diffusion metrics, (ii) included non-human models, (iii) lacked sufficient methodological detail regarding MRI acquisition or processing, or (iv) did not provide original data (e.g., reviews, editorials). Additionally, studies with overlapping cohorts or insufficient statistical reporting were excluded to ensure consistency and interpretability of findings (Figure 2).

The abbreviations used throughout the manuscript are summarized in Table 1 for reference. The principal findings of the included studies are summarized in Table 2, which outlines the key findings together with key MRI/DTI acquisition characteristics and complementary measures. The complete dataset is publicly available in the Open Science Framework (OSF) repository (https://osf.io/4rytn (DOI: https://doi.org/10.17605/OSF.IO/4RYTN); accessed on 8 June 2026).

Given that the primary objective of this review was to evaluate the applicability of the ALPS index, the strength and consistency of its associations with clinical and biological outcomes are a critical consideration. Across studies, reduced ALPS index values were consistently associated with cognitive impairment, Aβ burden, or disease staging [9,10,11]. For example, Huang et al. reported a correlation between ALPS index reduction and amyloid PET load [9], while Liang et al. found significantly lower ALPS values in persons with AD compared with controls [12], with values correlating with Clinical Dementia Rating (CDR) scores. Across studies, associations between lower ALPS index values and cognitive or pathological markers were generally moderate in magnitude, with several studies reporting significant correlations between ALPS measurements, amyloid burden, and neuropsychological performance scores. Collectively, these findings support the ALPS index as a potentially sensitive marker of glymphatic dysfunction in neurodegenerative disease contexts.

Methodological convergence across studies was observed. Most studies employed 3 Tesla MRI systems, except for the meta-analytic dataset reported by Costa et al., which included both 1.5T and 3T acquisitions [8]. Diffusion tensor imaging protocols were broadly consistent, typically using b values of 1000 s/mm^2^ and 30–64 diffusion directions [9,12,13,14]. Regions of interest (ROIs) were consistently defined adjacent to projection and association fibers near perivascular spaces, although variability in manual versus semi-automated placement was reported [9,12,15]. Importantly, some studies also controlled for factors such as head orientation and time of day of MRI acquisition, with Han et al. demonstrating time-of-day effects on glymphatic activity, underscoring the importance of temporal standardization of scanning in future protocols [13].

Several studies strengthened the biological plausibility of ALPS by integrating ALPS measurements with additional biomarkers. For example, Huang et al. and Hong et al. incorporated amyloid PET imaging [9,14]. Hsu et al. further included FDG-PET and cognitive assessments [11]. These multimodal approaches enable cross-validation across structural, functional, and biochemical domains, enhancing confidence in the validity of ALPS as a proxy of glymphatic function. In contrast, studies relying on structural analyses alone, such as Siow et al., highlight the associations and physiological interplay between ALPS values, sleep quality, and cognitive performance, extending its relevance beyond overt neurodegeneration [15].

An important and emerging theme across studies is the modulation of ALPS by physiological and genetic factors. Evidence from Siow et al. [15] and Han et al. [13] suggests that sleep quality, vascular health, and circadian rhythms influence glymphatic efficiency. Liu et al. [16] further demonstrated that AQP4 polymorphisms are associated with variations in ALPS and cognitive outcomes, providing genetic support for glymphatic mechanisms. Wright et al. [17] explored methodological variability, demonstrating the sensitivity of ALPS measurements to head positioning and diffusion orientation, reinforcing the need for standardized acquisition and processing guidelines.

The inclusion of Costa et al. is particularly noteworthy, as it represented the first Bayesian meta-analysis in this domain [10]. This study not only demonstrated the reproducibility of ALPS-related alterations across multiple datasets but also emphasized the importance of explicitly accounting for uncertainty and between-study heterogeneity. The application of advanced statistical frameworks, such as Bayesian approaches, enhances the robustness and reliability of emerging evidence for ALPS and reflects increasing methodological sophistication within the field.

An important unresolved question concerns the disease specificity of the ALPS index. While most studies included in this review focused on AD, emerging evidence suggests that alterations in ALPS may also be present in other neurodegenerative conditions, including Parkinson’s disease and related synucleinopathies, as well as in tauopathies and TDP-43 proteinopathies. For instance, differences in the ALPS index have been observed across both AD and Parkinsonian populations, indicating that glymphatic dysfunction may represent a broader feature of neurodegeneration rather than a disease-specific marker [10].

Beyond AD, altered ALPS-related diffusion patterns have also been described in Parkinson’s disease, cerebral small vessel disease, and other neuroinflammatory conditions. These findings suggest that DTI-ALPS abnormalities may reflect broader disturbances in perivascular fluid dynamics, vascular integrity, or neuroinflammatory activity rather than disease-specific pathological mechanisms.

In addition, alterations in perivascular diffusion metrics have been reported in non-neurodegenerative conditions, such as cerebral small vessel disease and inflammatory disorders of the central nervous system, including multiple sclerosis. These findings raise the possibility that the ALPS index may reflect global disturbances in interstitial fluid dynamics, vascular integrity, or neuroinflammatory processes.

Therefore, although current evidence supports the sensitivity of ALPS to pathological changes, its specificity for AD remains uncertain. This profile resembles other fluid-based biomarkers, such as neurofilament light chain, which are sensitive to neuroaxonal injury but not disease-specific. Future studies directly comparing multiple neurodegenerative and neuroinflammatory conditions using standardized protocols will be essential to clarify whether ALPS alterations are disease-specific or represent a common pathway of brain dysfunction.

At the molecular level, glymphatic function is critically dependent on the polarized expression of aquaporin-4 (AQP4) water channels located in astrocytic endfeet surrounding cerebral vasculature. AQP4 facilitates the convective exchange between cerebrospinal fluid (CSF) and interstitial fluid (ISF), thereby modulating solute clearance, including amyloid-β and tau proteins. Disruption of AQP4 polarization, frequently observed in aging and neurodegenerative diseases, has been associated with impaired glymphatic transport efficiency.

Neuroinflammatory processes, characterized by microglial activation and astrocytic reactivity, may alter perivascular space integrity and extracellular matrix composition, further affecting interstitial fluid dynamics. These processes may influence diffusion tensor imaging metrics, including the ALPS index, by modifying tissue microstructure and water diffusivity.

Vascular factors also play a central role. Arterial pulsatility is considered a driving force for glymphatic flow, and alterations in vascular compliance, commonly observed in cerebral small vessel disease, may impair perivascular fluid movement. Moreover, blood–brain barrier dysfunction may exacerbate interstitial fluid imbalance, contributing to altered diffusion patterns detected by DTI-ALPS.

An additional factor insufficiently explored in the current literature is the potential influence of biological sex on glymphatic-related processes and ALPS-derived metrics. Emerging evidence suggests that hormonal status, vascular physiology, sleep architecture, and neuroinflammatory responses may differ between males and females and could influence cerebrospinal fluid dynamics and interstitial fluid exchange. However, most currently available DTI-ALPS studies were not adequately powered to investigate sex-stratified effects.

Finally, emerging evidence suggests that genetic variability, particularly in AQP4-related polymorphisms, may modulate glymphatic efficiency and influence ALPS-derived metrics. These molecular and physiological mechanisms provide a biological substrate linking DTI-derived diffusion properties to glymphatic system function, although the ALPS index remains an indirect measure and should be interpreted within this broader mechanistic context.

Compared with established biomarkers such as amyloid positron emission tomography, tau positron emission tomography, cerebrospinal fluid biomarkers, and structural magnetic resonance imaging, DTI-ALPS offers the advantage of being non-invasive and relatively accessible within conventional MRI protocols. However, unlike molecular biomarkers with greater pathological specificity, ALPS-derived metrics remain indirect and may be influenced by multiple physiological and technical factors. Therefore, DTI-ALPS should currently be considered a complementary and exploratory biomarker rather than a standalone diagnostic tool.

Despite these advances, several limitations warrant consideration. First, sample sizes in individual studies were generally modest, limiting statistical power and generalizability. Second, heterogeneity in ROI placement, post-processing pipelines, and complementary imaging techniques reduces comparability across studies. Third, key confounders, including participant sleep patterns, vascular risk factors, and diurnal variation in MRI acquisition, were inconsistently accounted for, despite growing evidence of their influence [11,12]. Finally, while meta-analytic approaches such as those employed by Costa et al. [8] represent important methodological progress, broader adoption of multicenter, harmonized, and longitudinal approaches is essential to establish the ALPS index as a reliable clinical biomarker. Furthermore, the review process itself has limitations, including the search being restricted to two main databases and the exclusion of non-English or non-peer-reviewed literature, which may have omitted relevant preliminary data. An additional limitation of this review is that the literature search was restricted to the PubMed and Web of Science databases. Although these databases capture a substantial proportion of biomedical literature, relevant studies indexed in other databases such as Scopus or Embase may not have been identified.

Since the completion of the literature search for the present review, the DTI-ALPS field has continued to expand across a wide range of neurological and physiological contexts. Recent studies have extended the application of DTI-ALPS beyond AD and mild cognitive impairment to conditions including Lewy body disease, isolated REM sleep behavior disorder, multiple sclerosis, glioma, and vascular risk populations. In Lewy body disease, reduced ALPS values have been associated with cognitive decline and circulating biomarkers of neuronal injury, including neurofilament light chain and glial fibrillary acidic protein [6]. Similarly, lower ALPS values have been reported in isolated REM sleep behavior disorder and were associated with cognitive performance and regional brain atrophy, suggesting that glymphatic-related alterations may already be present during prodromal stages of α-synucleinopathies [18]. Longitudinal evidence from multiple sclerosis further suggests that lower baseline ALPS values may be associated with subsequent disability progression, supporting the potential prognostic value of ALPS-derived metrics [19].

Recent investigations have also explored physiological and vascular factors that may influence ALPS-derived measurements. Experimental sleep studies demonstrated significant increases in DTI-ALPS values during sleep compared with wakefulness, supporting the proposed relationship between sleep-dependent physiological processes and glymphatic-related fluid dynamics [20]. In addition, a large population-based cohort study reported associations between prolonged exposure to elevated diastolic blood pressure and lower ALPS values, highlighting the potential contribution of vascular health to glymphatic-related imaging markers [7].

At the same time, several publications have focused on methodological validation and biological interpretation of DTI-ALPS measurements. Comparative analyses between DTI-ALPS and intrathecal contrast-enhanced MRI demonstrated only limited associations between ALPS-derived measurements and direct tracer-based markers of cerebrospinal fluid dynamics, reinforcing ongoing concerns regarding the biological specificity of the ALPS index [21]. Similarly, investigations performed using 1.5T MRI systems identified substantial inter-rater variability and emphasized the need for standardized ROI placement and harmonized image-processing protocols [2].

Beyond original research studies, several reviews and perspective articles have synthesized emerging knowledge regarding glymphatic physiology, the role of AQP4, sleep-dependent fluid transport, meningeal lymphatic pathways, and current approaches for assessing glymphatic function in humans [22]. Collectively, these publications highlight both the growing interest in glymphatic-related neuroimaging and the continuing uncertainties surrounding the physiological mechanisms captured by DTI-ALPS measurements.

Taken together, these recent developments illustrate the rapid evolution of the field and reinforce the need for multicenter longitudinal investigations, standardized acquisition and processing pipelines, and multimodal validation studies capable of clarifying the biological significance and clinical applicability of ALPS-derived metrics.

## 4. Conclusions

The DTI-ALPS approach represents a promising non-invasive neuroimaging strategy for investigating diffusion alterations potentially related to glymphatic-associated processes in neurodegenerative diseases. Nevertheless, current evidence remains preliminary and limited by methodological heterogeneity, modest sample sizes, and unresolved physiological uncertainties regarding the interpretation of diffusivity metrics as indicators of fluid transport. Although several studies demonstrated associations between ALPS-derived measurements and cognitive or pathological markers, the biological specificity and clinical applicability of this method remain insufficiently established. Future multicenter longitudinal studies employing harmonized acquisition protocols, standardized processing pipelines, and multimodal biomarker integration will be essential to determine the translational utility of DTI-ALPS in neurodegenerative disease research.

## Figures and Tables

**Table 1:** List of abbreviations used throughout the manuscript.

Abbreviation	Full Name
AD	Alzheimer’s Disease
ALPS	Analysis along the Perivascular Space
ANOVA	Analysis of variance
ANCOVA	Analysis of covariance
AQP4	Aquaporin-4
CDR	Clinical Dementia Rating
CSF	Cerebrospinal fluid
CSVD	Cerebral small vessel disease
DTI	Diffusion tensor imaging
FA	Fractional anisotropy
FDG-PET	Fluorodeoxyglucose positron emission tomography
FSL	FMRIB Software Library
MCI	Mild cognitive impairment
MD	Mean diffusivity
MRI	Magnetic resonance imaging
PD	Parkinson’s disease
PET	Positron emission tomography
ROI	Region of interest
SPM	Statistical parametric mapping
T	Tesla

**Table 2:** Summary of included studies.

Study	Population	MRI/DTI Protocol	Additional Measures	Key Findings
Huang et al., 2024	Population: *n* = 100 (AD, controls)	MRI: 3T; DTI: b = 1000, 32 directions	Metrics: ALPS index, FA Structural MRI, Amyloid PET; Processing: SPM, DSI Studio; Statistics: ANOVA, regression	Lower ALPS index values were associated with higher amyloid PET burden and worse cognitive performance
Sacchi et al., 2024	Population: *n* = 86 (MCI, AD)	MRI: 3T; DTI: b = 1000, 64 directions	Metrics: ALPS index, MD CSF biomarkers (AQP4); Processing: FSL, custom ALPS pipeline; Statistics: *t*-test, regression	ALPS index values were associated with CSF AQP4 concentrations
Costa et al., 2024	Population: Meta-analysis (*n* = 362 total; AD, PD, controls)	MRI: 1.5T and 3T; DTI: varied	Metrics: ALPS index; Processing: Meta-analytic harmonization; Statistics: Bayesian meta-analysis	ALPS index values differed between clinical and control groups across datasets
Liang et al., 2023	Population: *n* = 60 (AD vs. controls)	MRI: 3T; DTI: b = 1000, 30 directions	Metrics: ALPS index, FA; Additional: T1-weighted MRI; Processing: DTI Toolkit + MATLAB (V. R2023a); Statistics: *t*-test, ANOVA	ALPS index values were lower in AD compared to controls and correlated with CDR scores
Siow et al., 2021	Population: *n* = 84 (community elderly)	MRI: 3T; DTI: b = 1000, 30 directions	Metrics: ALPS index; Additional: Cognitive testing, sleep studies; Processing: Standard DTI preprocessing; Statistics: Correlation	ALPS index values were associated with sleep quality and cognitive performance
Hong et al., 2024	Population: *n* = 105 (Preclinical AD, MCI, AD, controls)	MRI: 3T; DTI: b = 1000, 64 directions	Metrics: ALPS index; Additional: CSF markers, amyloid PET; Processing: FSL, FreeSurfer; Statistics: Multivariate regression	ALPS index values were associated with amyloid burden and markers of cerebral small vessel disease
Han et al., 2023	Population: *n* = 50 (healthy)	MRI: 3T; DTI: b = 1000, 60 directions	Metrics: ALPS index; Processing: DTIStudio; Statistics: Mixed-effects modeling	ALPS index values varied according to age and time of day
Wright et al., 2023	Population: Methodological study (healthy, MCI)	MRI: 3T; DTI: b = 1000, 30 directions	Metrics: ALPS index, FA; Processing: Comparative DTI modelling; Statistics: Technical validation tests	ALPS measurements were influenced by head orientation and diffusion directionality
Liu et al., 2025	Population: *n* = 150 (aging cohort)	MRI: 3T; DTI: b = 1000, 64 directions	Metrics: Free water imaging, ALPS index; Additional: Genetic analysis (AQP4 SNPs); Processing: FreeSurfer + DTI pipeline; Statistics: Multivariable regression	AQP4 genetic variants were associated with diffusion measures and cognitive outcomes
Hsu et al., 2024	Population: *n* = 98 (AD, controls)	MRI: 3T; DTI: b = 1000, 32 directions	Metrics: ALPS index, MD; Additional: FDG-PET, neuropsych testing; Processing: SPM12; Statistics: ANCOVA, correlation	Lower ALPS index values were associated with worse cognitive performance

## Data Availability

The data supporting the findings of this study are publicly available in the Open Science Framework (OSF) repository at: https://osf.io/4rytn (DOI: https://doi.org/10.17605/OSF.IO/4RYTN) (accessed on 8 June 2026). The repository contains the full extracted dataset, a structured study-characteristics table (Appendix A), and supporting materials used for data synthesis and visualization.

## Appendix A. Database Search Strategies

PubMed Search Strategy

(
“Neurodegenerative Diseases”[MeSH]
OR “Alzheimer Disease”[MeSH]
OR “Cognitive Dysfunction”[MeSH]
OR “Dementia”[MeSH]
OR “Mild cognitive impairment”[tiab]
)
AND
(
“Aquaporin 4”[MeSH]
OR “Aquaporins”[MeSH]
OR “Cerebrospinal Fluid”[MeSH]
OR “Perivascular Spaces”[tiab]
OR “Glymphatic System”[tiab]
OR “Brain Edema”[MeSH]
)
AND
(
“Diffusion Tensor Imaging”[MeSH]
OR “Diffusion Magnetic Resonance Imaging”[tiab]
OR “Image Interpretation, Computer-Assisted”[MeSH]
OR “Neuroimaging”[MeSH]
)

Filters Applied: Publication dates from 2010 to 2025
Results Retrieved: 158 records

Web of Science Core Collection (WoSCC) Search Strategy

(
“Alzheimer Disease”
OR “Neurodegenerative Diseases”
OR “Cognitive Dysfunction”
OR “Dementia”
OR “Mild Cognitive Impairment”
)
AND
(
“Glymphatic System”
OR “Perivascular Spaces”
OR “Cerebrospinal Fluid Circulation”
)
AND
(
“Diffusion Tensor Imaging”
OR “DTI”
OR “Diffusion MRI”
OR “Magnetic Resonance Imaging”
)
AND
(
“Human”
OR “Humans”
)

Filters Applied: Publication years 2010–2025; Open Access only
Results Retrieved: 94 records
